# Truck Travel times on freeway facilities and segments in Germany based on FCD from 2019

**DOI:** 10.1038/s41597-026-07198-z

**Published:** 2026-04-08

**Authors:** Marian Schlott, Lateef Abdul, Bert Leerkamp

**Affiliations:** University of Wuppertal, Wuppertal, Germany

**Keywords:** Research data, Geography

## Abstract

The paper describes the generation of truck travel times using Floating Car Data (FCD) for the year 2019 on German freeway facilities. FCD, collected by German Automobile Club (ADAC), include over 25 billion data points, which are assigned to a network model using the OpenSourceRoutingMachine (OSRM) map matching service. A k-means clustering algorithm classifies vehicles by type (e.g. car or truck) based on their velocity profiles. The Adaptive Smoothing Method (ASM) is applied for spatiotemporal interpolation, improving velocity estimates and deriving a continuous velocity function. The final method calculates travel times for predefined segments by adjusting velocities based on FCD-derived traffic conditions dynamically, updating every 10 minutes to reflect current traffic conditions. This approach provides detailed and reliable estimates of truck travel times, with a focus on accuracy through sufficient data penetration and aggregation intervals.

## Background & Summary

The unreliability of truck travel times on freeway facilities has significant impacts on processes in the supply chain, including:

- Route optimisation and route planning for truck fleets,
- Capacity management at loading

Evidently, trucks on highway facilities experience significantly greater travel time losses due to disruptions or overloading of the infrastructure compared to passenger cars. These losses cannot be compensated on subsequent network segments due to the maximum permissible speed of 80 kilometers per hour (kph) for trucks in Germany^1^.

The unreliability of truck traffic affects not only a single stakeholder in the supply chain (e.g., the shipper) but also impacts the logistical decisions of various actors and stages within the supply chain^2^. To minimize the impacts on various actors, the accurate prediction of travel times in motorway networks is essential. Customer demands for modern logistics solutions, on the one hand, and the need to optimize the efficiency of transport resources, on the other, make it necessary to forecast travel times as reliably as possible, including accounting for delays caused by traffic flow disruptions. However, publicly available travel time data (esp. for trucks) are rare.

We utilize Floating Car Data (FCD) to estimate travel times for trucks on freeway facilities and segments in Germany in 2019.

The datasets provided, contribute to enhancing the prediction of travel times on German highways and serve as a basis for analysing truck travel time reliability on an annual scale. These datasets include a network model that differentiates between highway segments and facilities, as well as travel times for weekdays (Monday to Friday) for trucks in 2019. Travel times has been computed for all freeway facilities and their segments between 04:00 and 20:00. Calculations has been made at ten-minute intervals across all weekdays (Monday to Friday). The data contributes to a better understanding of truck traffic on (German) highways and could be used in various tasks.

## Methods

In the following, the general process of generating the travel times are laid out starting with the network model shortly; after that we describe the data processing of the Floating Car Data.

### Network model

The network model, obtained from OpenStreetMap (OSM) for the year 2019, has been segmented to differentiate three aggregate levels^3^. The smallest aggregate level is the 100 m-edge which is the basis for the network assignment algorithm; so the 100 m-edge serves as the aggregation level for the FCD to secure a certain spatial resolution and plays also a crucial role for the interpolation algorithm conducted.

**Fig. 1:**
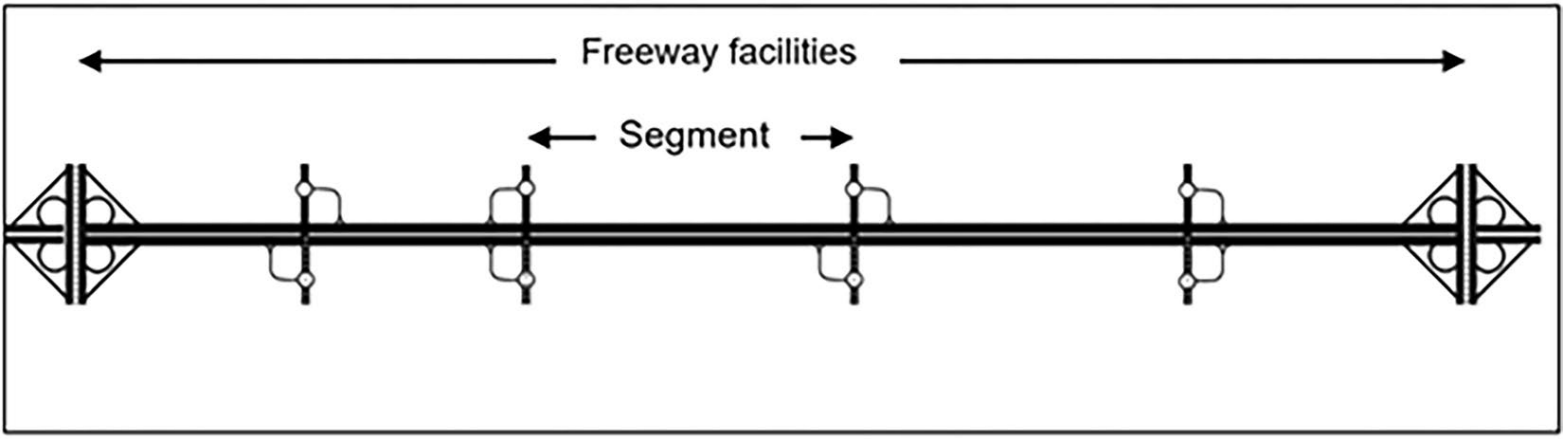
Schematic representation of freeway facilities on highways.

In general, freeway facilities comprise basic, diverge, merge and weaving segments or intersections^4,5^. To enable the analysis of travel times at both segment and freeway facility level, an algorithm was developed to segment the network model. The algorithm requires the classification of various motorway elements, including basic, diverging, merging, and weaving segments, as well as intersections. Based on this classification, 100-meter-edges are aggregated until they intersect with elements classified as basic, diverging, merging, or weaving segments. The freeway facilities were derived from the highway network classification of the federal ministry of Digital and Transport. (https://bmdv.bund.de/SharedDocs/DE/Artikel/StB/Verbindungsfunktionsstufen-0-1.html) Fig. 1depicts the distinction between freeway facilities and segments^6^. The figure illustrates the hierarchical structure of a freeway facility, comprising multiple segments.

### Processing of floating car data

Floating Car Data (FCD) is generated by on-board units or navigation systems in vehicles and provides information on vehicle velocity over time (timestamp) and space (geocoordinates). Data providers typically offer either ‘raw’ FCD or aggregated FCD, such as percentiles of velocities over specific road network elements. ‘Raw’ FCD, in this context, refer to data that can either track an individual vehicle’s movement over time or be aggregated over specific network elements to provide an overview of traffic conditions. We utilize ‘raw’ FCD data for the year 2019, provided by German Automobile Club (ADAC). The ADAC collects FCD from various providers to monitor and estimate traffic conditions. We do not have any specific information on the providers from which the ADAC collects the data. Our sample comprises more than 25 billion data points. The number of FCD hits varies between months; approximately 2 billion FCD hits are recorded each month, with notably higher numbers in the summer months, which can be attributed to holiday traffic. The hourly distribution of FCD hits is relatively consistent across months and days, suggesting that the composition of the FCD sample is primarily influenced by fleets of major logistics service providers. Checking the daily distribution of the sample, we can observe the formation of a plateau which is more common in truck traffic unlike to the observed daily patterns in passenger traffic, with two peaks during the morning and evening rush hours. In addition to the number of FCD hits and their hourly distribution, the examination of individual vehicles is of interest. For anonymization reasons, a vehicle can only be tracked in the FCD sample for 24 hours^7^. Given that we are using a raw FCD sample, we need to conduct a network assignment as well as a vehicle classification.

### Network assignment

To compute travel times, it is necessary to assign the FCD to the network model. For this study, the map matching service of the OpenSourceRoutingMachine (OSRM) is used^8^. OSRM is an open-source router offering various services based on OSM data, utilizing a Hidden Markov Model (HMM) for map matching. HMMs determine the probability of reaching a state B from an initial state A. In map matching, HMMs evaluate the likelihood that a sequence of road elements forms the actual route of a vehicle trajectory. The states in the HMM are network elements, while the state observations are FCD points dispersed spatially and temporally. The goal of the HMM is to assign the FCD to the correct network element with high probability. (plausibility)^9^. Due to the high cost of map matching services, an OSRM instance is initiated in a Docker environment. The required inputs include e.g. GPS coordinates, timestamps, directions, and a maximum search radius per vehicle, set at 25 meters due to the computational intensity of the network assignment and suitabiltiy for the assignment to highways. OSRM returns the corresponding OSM node. We then use the respected OSM node for the assigning to our network model (100m-edge). The methodology prevents “noisy” FCD hits from being assigned to a network edge, reducing the need for extensive plausibility checks. For instance, the algorithm verifies if the transmitted velocity is realistic for the specific road class.

### Vehicle classification

After the assignment we need to classify the vehicles to distinguish between cars and trucks. For that we choose a clustering approach; in that matter a vehicle database is prepared, capturing distribution parameters (minimum, maximum, median, and average velocity) for each vehicle on different road types (e.g., highways). A k-means clustering algorithm is applied, which in general partitions the data into a predefined number of clusters, k. Each cluster contains at least one object, and each object belongs to exactly one cluster. The goal is to minimize the sum of squared deviations from the cluster centroids. Initially, k centroids are randomly placed within the data points. The centroids are then recalculated as the mean of all members within each cluster, and data points are reassigned. This process repeats until no changes occur, reaching an optimal solution^10^. The FCD sample consists of around 38 million vehicle IDs (asset IDs) that transmitted signals on motorways and sent at least 50 hits. Crucially, each vehicle is only traceable for 24 hours — after which a new asset ID is assigned. This means that a misclassified vehicle cannot propagate classification errors across the entire study period; any error is limited to a single day’s observation.

**Fig. 2:**
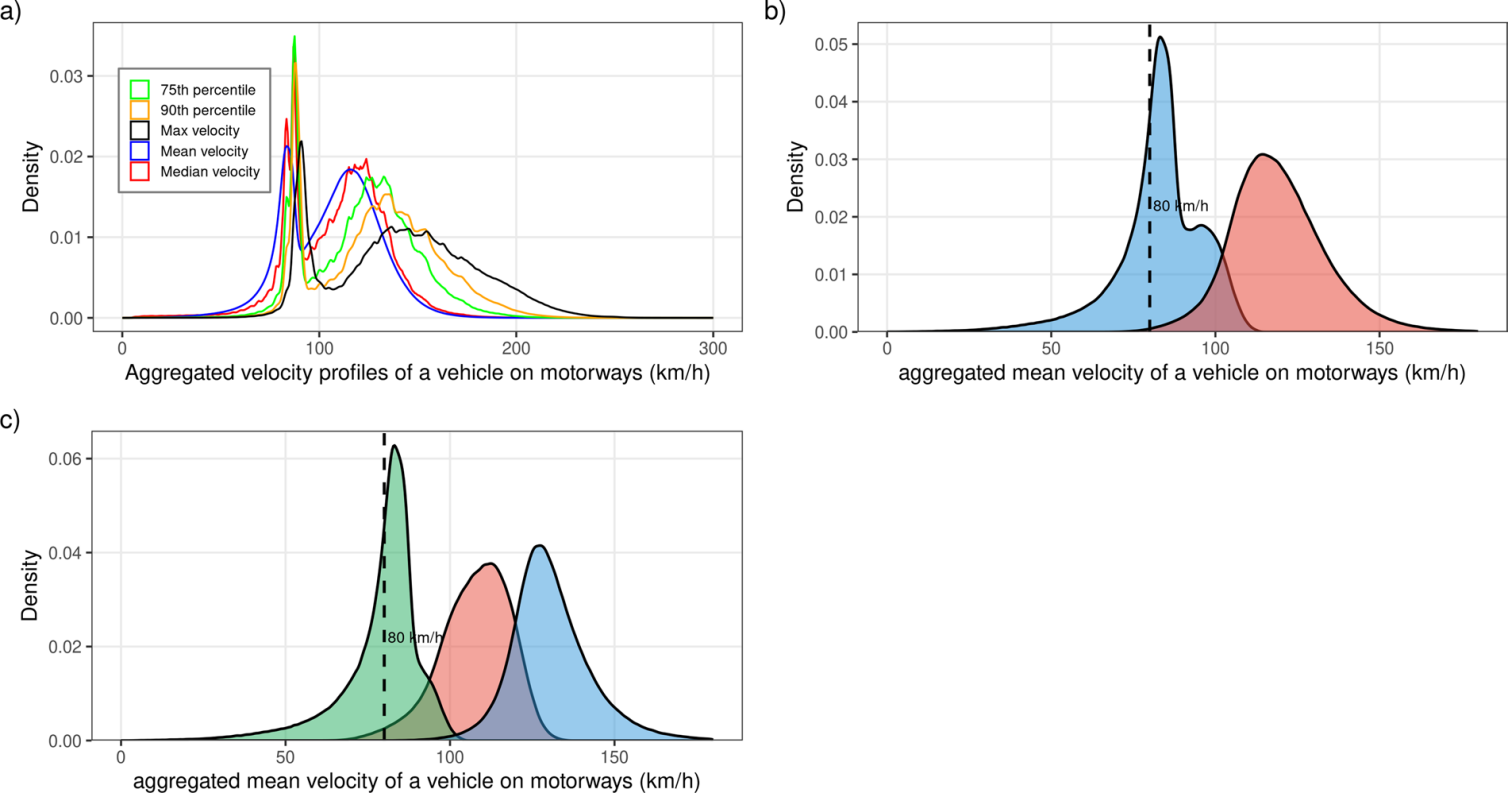
Density distributions of aggregated velocity profiles on motorways used as input for k-means clustering. (a), Density distributions of five velocity metrics derived from FCD for all vehicles on motorway segments ranging from 0 to 300 km/h. (b), Density distributions of aggregated mean velocity for two k-means derived vehicle clusters: trucks (blue) and passenger cars (pink). The dashed vertical line indicates the 80 km/h threshold, corresponding to the legal speed limit for trucks in Germany. c), Density distributions of aggregated mean velocity for three k-means derived vehicle clusters: trucks (green), intermediate cluster (pink), and passenger cars (blue). The dashed vertical line indicates the 80 km/h threshold.

As stated above, velocities on freeway segments are used as clustering inputs. We calculate for each vehicle ID with more than 50 hits on freeways different velocity profiles. The distribution of the aggregated velocity attributes shows two clear modes across all aggregation levels (see Fig. 2 Panel a), which is consistent with a two-class structure (trucks and cars). The bimodal nature of the distribution supports the use of velocity as the primary discriminating feature in this specific context.

We use three initial clusters for the k-means algorithm instead. We acknowledge that the silhouette score is lower for k = 3 (0.489) than for k = 2 (0.553), and that this could be interpreted as arguing in favor of a two-cluster solution. However, the choice of k = 3 was deliberate and methodologically motivated.

As shown in Fig. 2 panel a), the velocity distribution exhibits two dominant peaks, which at first glance does not clearly suggest the presence of three clusters. However, the transition zone between the truck and car distributions which likely correspond to congested freeway conditions where car and truck speeds converge, creates a region of potential misclassification. Therefore, vehicles in this overlap zone (possibly congested cars or buses) could incorrectly be assigned to the truck cluster under k = 2 (Fig. 2 panel b)).

By using k = 3 (Fig. 2 panel c)), we introduce a third cluster that captures this intermediate velocity profile. Cars from the transition zone are then assigned into either the intermediate or the car cluster, reducing false assignment to the truck cluster. Since the study’s primary focus is on heavy goods vehicles (>7.5 t gross weight), we deliberately accept a smaller truck sample size in exchange for a cleaner, less noisy truck classification. In other words: we prefer a smaller truck sample over a larger but more inaccurate one.

### Dynamic travel times

To reflect actual velocities (ground truth) on segments and freeway facilities, it is essential to determine the number of FCD hits required within a specific time interval to reliably estimate the velocity. The first step in this process involves evaluating the penetration rate of the FCD sample at counting stations operated by the Federal Highway Research Institute (BASt) over an entire year. (https://www.bast.de/DE/Themen/Digitales/HF_1/Massnahmen/verkehrszaehlung/Stundenwerte.html?nn=414410) On average, penetration rates of approximately 5.7% for trucks and 2.3% for passenger cars are derived by comparing the hourly traffic volumes recorded at the counting stations with the FCD-based traffic data. For hourly evaluations, the penetration rate is sufficient. A penetration rate of 2% is adequate to reliably provide information about traffic conditions^11^. However, hourly travel time distributions are too “coarse” to accurately capture traffic conditions. In the literature it is recommended to aggregate travel times or velocities within time intervals of no more than 15 minutes for traffic condition estimation using FCD^12^. To evaluate penetration rates for different time intervals, we analyse data from two counting stations in North Rhine-Westphalia (NRW) for which traffic data (vehicle volumes and velocities for cars and trucks) are available at 1-minute intervals over an entire year. For the traffic station MQ_46.14a_HFB_SW, the average monthly penetration rate ranges between 1.9% and 2.5%. In contrast, at the traffic station MQ_46.16a_HFB_NO, the average monthly penetration rate spans between 4.4% and 7.3%. To estimate the accuracy of velocities derived from FCD, we compare FCD-based velocities with those measured at traffic stations for various time intervals and numbers of hits. To directly relate the analysis to the measured velocity data, we apply the following methodology outlined in^13^. This approach utilizes the Standard Quality Value (SQV) metric to evaluate the quality of velocities derived from different numbers of vehicle trajectories.

The SQV is calculated as:

$${g}_{{SQV}}=\frac{1}{1+\sqrt{\frac{{\left(m-c\right)}^{2}}{f\ast c}}}$$

where m represents the measured velocity, c is the reference velocity, and f is a scaling factor^14^. The scaling factor f is determined based on the typical magnitudes of the evaluated traffic conditions, taking into account velocities of 80 kph for trucks.

**Fig. 3:**
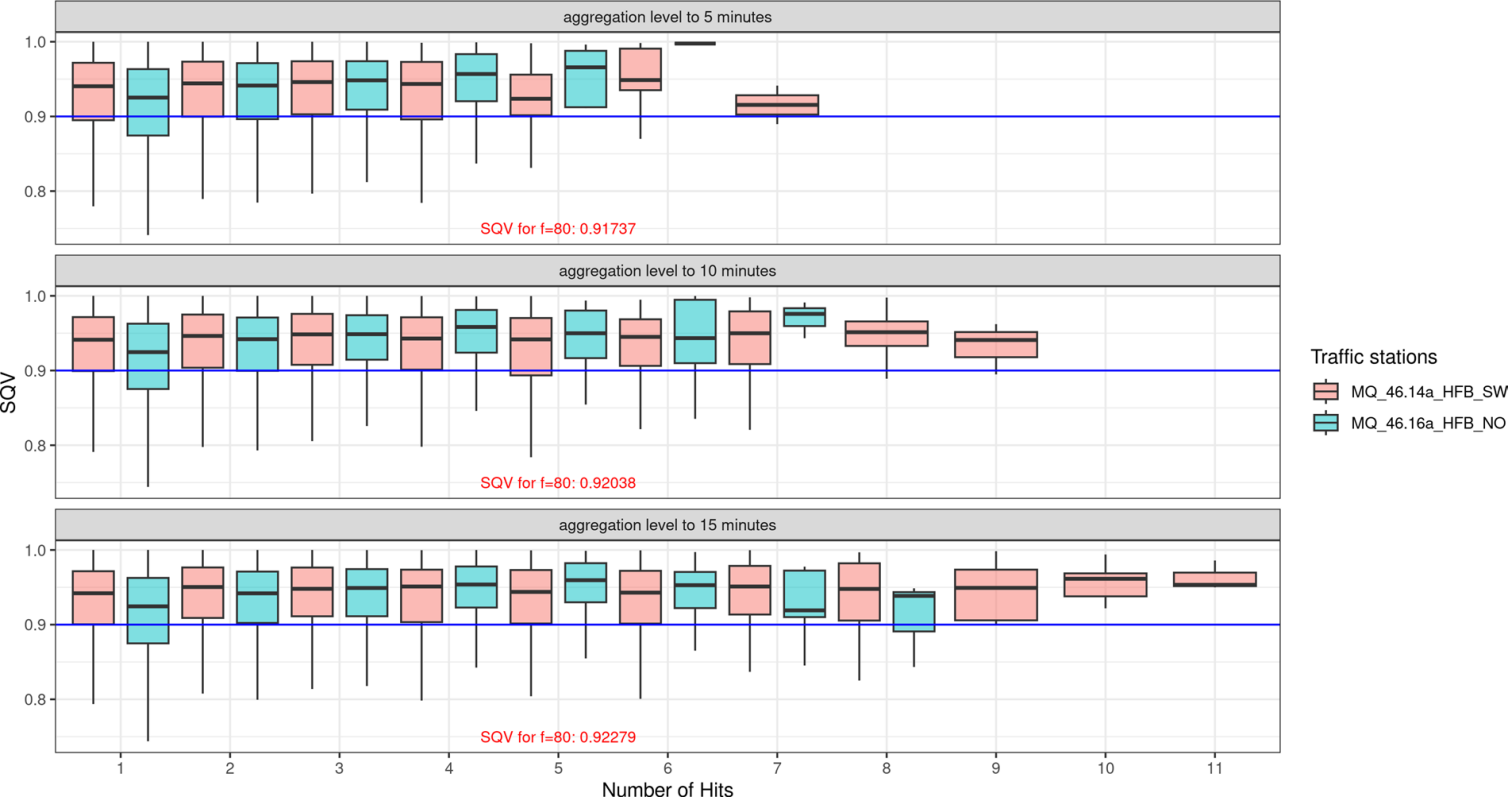
SQV for truck velocities from FCD vs. velocities measured on traffic stations across different aggregation levels and number of FCD hits; Box plots showing the distribution of SQV values as a function of the number of FCD hits (1–11) for two traffic stations (MQ_46.14a_HFB_SW, pink; MQ_46.16a_HFB_NO, teal) at aggregation levels of 5 minutes, 10 minutes, and 15 minutes. The horizontal blue line indicates the SQV threshold of 0.9, representing a very good match between compared values.

The SQV metric measures the quality of the velocity estimates derived from FCD compared to ground truth data (traffic station velocities). A higher SQV indicates better agreement, with an SQV of 0.9 or above considered a “very good match”^14,15^. We aggregate the velocity measured on traffic stations by computing the harmonic mean for different time levels. For all aggregation levels (5, 10, and 15 minutes), the SQV improves with an increasing number of hits. The average SQV consistently exceeds the 0.9 threshold across all levels, indicating a strong match between FCD-derived and ground truth velocities. Both traffic stations (MQ_46.14a_HFB_SW and MQ_46.16a_HFB_NO) show similar trends, with some variability. Notably, for lower hit counts, SQV at MQ_46.16a_HFB_NO tends to be slightly higher than at MQ_46.14a_HFB_SW, suggesting marginally better performance at the former station. Longer aggregation levels slightly enhance SQV but may sacrifice temporal resolution. For instance, a 15-minute aggregation yields higher SQV but may be less responsive to short-term traffic fluctuations. Overall, the Fig. 3 demonstrates that an SQV value of 0.9 or higher is achievable with adequate FCD hits, even with shorter intervals like 5 minutes. These findings validate the use of FCD for velocity estimation, particularly when penetration rates are sufficient.

The analysis shown in Fig. 3 indicates that a 10-minute aggregation level offers a high temporal resolution for assessing truck travel time reliability using the available FCD sample. Given the spatial and temporal variability in FCD penetration rates, interpolation is necessary to derive a continuous velocity function for specific time intervals and spatial resolutions. The sample includes approximately 19 truck FCD hits per kilometre of freeway per hour, which is insufficient to detect traffic disruptions and their temporal and spatial fluctuations. Therefore, the Adaptive Smoothing Method (ASM)^16,17^ is applied. Spatiotemporal interpolation algorithms provide continuous average velocities as functions of space and time, derived from traffic stations. Since the FCD sample is assigned to the network model, they can be spatially and temporally aggregated, making the methodology applicable to the available data^16,17^.

**Fig. 4:**
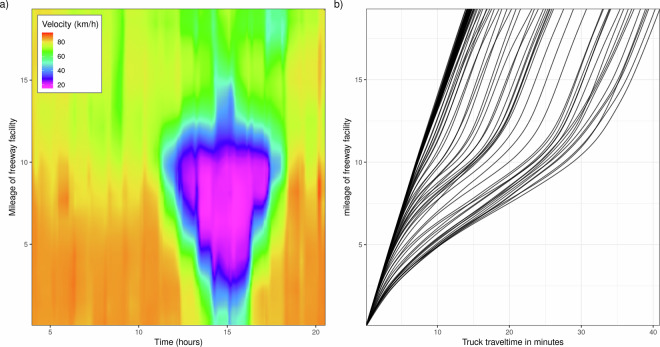
Spatiotemporal velocity distribution and derived truck travel times along a freeway facility. (a), heatmap showing the continuous velocity field (km/h) across the freeway facility, derived using the ASM method. The x-axis represents time of day (hours) and the y-axis represents the mileage along the freeway facility (km). (b), Trajectory lines showing derived truck travel times (minutes) as a function of entry position along the freeway facility. Each line represents a distinct departure time interval; diverging trajectories indicate increasing travel time delay caused by the congestion event visible in a.

Figure 4 panel a) illustrates the ASM-method results, showing a central region from 10:00 to 17:00 with a significant red zone indicating reduced truck velocity due to congestion or other factors, suggesting a peak traffic impact during this period. So the implemented method reflects real world congestion on a temporal and spatial high resolution level. We have applied this method to all freeways during the time intervals from 04:00 to 20:00, generating synthesized velocities for each 100-meter segment at ten-minute intervals. The final step is then to compute the travel times; for that we implement the following method: As the path of a segment or freeway facility is predefined, we start at the first 100m-edge of a segment or freeway facility and end at the last 100m-edge, along with a particular start time—for example, 08:00 hours (this can be adjusted based on requirements). Starting from the initial time, as the segments and freeway facilities are divided into 100m-edges, we calculate the travel time for each 100m-edge at the starting time, keeping track of the cumulative travel time spent as we move from one 100m-edge to the next. This process continues until the total time reaches 10 minutes (assuming the last 100m-edge has not yet been reached). At this point, we update the velocity to reflect the conditions at 08:10 hours and repeat the process for the next 10-minute interval. This cycle of updating the velocity every 10 minutes and calculating travel times for each 100 m-edge continues until the last element is reached leading to dynamic travel times for trucks. Finally, we have a plot (Fig. 4 panel b)) illustrating how travel time is affected temporarily and spatially considered in the method.

## Data Records

The datasets are available at Zenodo repository^18^. The network model data set is stored in a CSV-format and named network_model_agg.csv. The truck travel time data are stored in parquet-format and named traveltimes_networksegments.parquet (freeway facilities) and traveltimes_segments.parquet (segments).

### Data definitions

#### Network model

The structure of the network model dataset is summarized in Table 1. The table describes the attributes used to represent freeway facilities and their segments.

**Table 1:** Schema of the network segment table describing the spatial structure of freeway facilities.

Column	Description
network_segment_id	Integer Identifier for the freeway facilities
segment_id	Integer Identifier for the segments
array_osm_id	Integer[] Array storing all osm_ids on the segment.
geom	MultiLineString (WGS 4326) Stored as Well-Known-Text

The network model provides a spatial assignment of travel times, including two key identifiers:

- Network Segment ID: Differentiates freeway facilities and enables the association of travel times with the network model (a.network_segment_id = b.network_segment_id).
- Segment ID: Refers to individual segments within a freeway facility, which may consist of multiple segments. Travel times can be linked at the segment level using (a.segment_id = b.segment_id).

The network model can be accessed through GIS systems, including all versions of QGIS, as well as standard geospatial libraries such as GeoPandas or sf. It was tested on QGIS 3.38.

### Truck Travel times

Truck travel times are categorized by freeway facilities and segments. Data is collected during weekday periods (Monday to Friday) in 10-minute intervals between 04:00 and 20:00.

The metadata structure for the truck travel time datasets is summarized in Table 2. We encourage to access the data using duckdb and to keep in mind that the timestamps are converted to Europe/Berlin. The parquet files can be accessed using libraries such as PyArrow and Pandas in Python, as well as Arrow and tidyverse extensions in R. It was tested on duckdb-version v1.5 using the CLI-client on Linux.

**Table 2:** Schema of the truck travel time datasets containing dynamically modelled travel times for trucks on German freeway facilities.

Column	Description
network_segment_id	Integer Identifier for the freeway facilities in the parquet-file truck_traveltimes_network_segments.parquet
segment_id	Integer Identifier for the segments in the parquet-fle truck_traveltimes_segments.parquet
timestamp	Timestamp with time zone (Europe/Berlin)
truck_traveltime_minutes	double dynamic travel times of trucks

The following table describes the Metadata for both parquet-files.

## Technical Validation

**Fig. 5:**
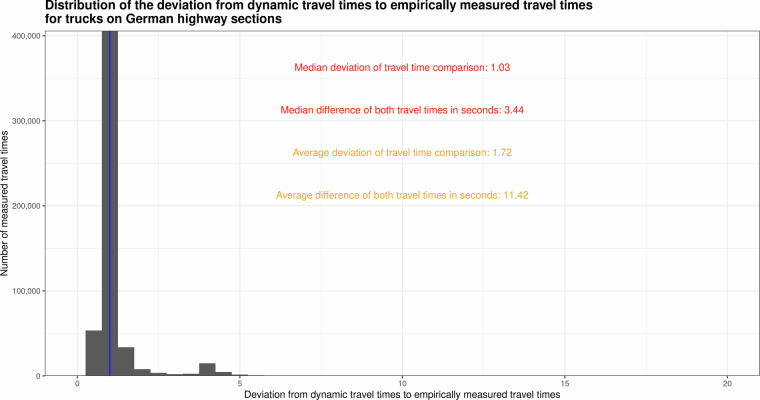
Distribution of deviations between dynamically modelled and empirically measured truck travel times on German freeway sections. Histogram showing the frequency distribution of the ratio between dynamic and empirically measured travel times across all evaluated truck trajectories on German highway sections. The vertical blue line indicates a deviation ratio of 1.0, representing perfect agreement between both methods.

We conduct a validation at each step of the data processing workflow (cp. to the sections). To validate the dynamic travel times we compare them against empirically measured travel times on section level. To derive empirically measured travel times, the FCD sample must first be segmented into individual trips per vehicle. This segmentation is carried out using a threshold-based approach, which defines the boundaries of a trip. Since this study focuses solely on travel times at the level of segments, extensive segmentation is not necessary. Only “segment-specific trips” need to be differentiated, meaning that when deriving travel times at the level of segments, it must be ensured that the derived travel times belong to a single trip. Consequently, FCD hits from a vehicle are assigned to the same trip as long as the time interval between consecutive FCD hits does not exceed 10 minutes. If this threshold is exceeded, the trip is considered to have ended. All vehicles that transmit an FCD hit within a trip on both the first and last section element are identified. The travel time of a vehicle on a specific segment of a trip is calculated by subtracting the timestamp of the first hit from that of the last hit. However, the average length of the first and last elements of a section is approximately 85.5 m, which must be considered. In some cases, the first hit may be recorded near the end of the first element, or the last hit may occur near the beginning of the last element. After that we have a sample of empirically measured travel times on sections. To compare both samples we compute the travel time share (dynamic travel times to empirically measured travel times) and the travel time difference. The distribution of the comparison between dynamic computed travel times and empirically measured travel times for trucks on German highway segments are depicted in the following Fig. 5. The histogram (Fig. 5) exhibits a highly skewed distribution, with the majority of the data concentrated near 1, indicating minimal deviation for most comparisons. A long tail extends towards larger comparison values, suggesting a smaller number of cases with significant deviations. The median deviation (1.03) suggests that dynamic computed travel times are close to empirically measured travel times for most cases as well as relatively low median difference (3.94 seconds) indicates good agreement between the two measures for travel times. The long tail in the distribution and the relatively high average difference (15.79 seconds compared to the median of 3.94 seconds) highlight the presence of outliers or cases with substantial deviations. The larger average values suggest that a subset of long sections with significant deviations is skewing the results, indicating that section length might play a key role. Nevertheless, the low median values demonstrate that the method for estimating travel times dynamic is reliable for the majority of cases.

Ground-truth validation of the classification is not available in this study, as access to vehicle registration data or OBU records containing explicit vehicle class labels was not feasible for the dataset used. Nevertheless, several indirect indicators support the plausibility of the classification. First, the velocity distribution of the derived truck cluster (Fig. 2, Panel a) closely reflects the known speed behavior of heavy goods vehicles on German motorways, where the legal speed limit is 80 km/h. The cluster’s median and mean speed (approximately 85 km/h) are consistent with the commonly observed slight exceedance of this limit typical for this vehicle class. Second, the temporal constraint of 24-hour asset ID validity limits the impact of any individual misclassification, since potential classification errors cannot persist beyond a single day. Third, the multi-stage framework further mitigates the influence of individual misclassified observations on the resulting aggregate travel time estimates. In a final step, vehicle-level observations are aggregated at the network level, further reducing the impact of remaining outliers (misclassified trucks) and yielding stable network-wide travel time estimates. In addition to these structural plausibility arguments, we conducted an indirect empirical validation by comparing FCD-derived truck speeds with speeds recorded at traffic count stations. The results in (Fig. 3) show that SQV values consistently exceed 0.9 across all aggregation levels and hit counts, reaching 0.917 (5 min), 0.920 (10 min), and 0.923 (15 min) at the reference point of f = 80. Since an SQV above 0.9 indicates very high agreement between the two data sources for the same time intervals, this finding strongly supports the validity of the truck classification. If a substantial proportion of vehicles in the truck cluster were in fact misclassified cars, the resulting speed distributions would diverge noticeably from the traffic station benchmark, which is not observed in the comparison.

## Usage Notes

As the timestamps are converted to Europe/Berlin and libraries such as pandas or duckdb often interpret timestamps as UTC, you must always execute a query to set the time zone to ‘Europe/Berlin’.

## Data Availability

The datasets generated during this study are available at Zenodo reposity^18^. There are no access restrictions to the data.

## Code availability

The code can be accessed using the following link: https://git.uni-wuppertal.de/schlott/truck-travel-times-on-freeway-facilities-and-segments-in-germany-based-on-fcd-from-2019.
